# Plasmid-mediated tigecycline-resistant gene *tet*(X4) in *Escherichia coli* from food-producing animals, China, 2008–2018

**DOI:** 10.1080/22221751.2019.1678367

**Published:** 2019-10-21

**Authors:** Chengtao Sun, Mingquan Cui, Shan Zhang, Hejia Wang, Li Song, Chunping Zhang, Qi Zhao, Dejun Liu, Yang Wang, Jianzhong Shen, Shixin Xu, Congming Wu

**Affiliations:** a Beijing Advanced Innovation Center for Food Nutrition and Human Health, College of Veterinary Medicine, China Agricultural University, Beijing, People’s Republic of China; b China Institute of Veterinary Drug Control, Beijing, People’s Republic of China

**Keywords:** Mobile tigecycline resistance, *Escherichia coli*, food-producing animals, retrospective analysis, China

## Abstract

The recent emergence of plasmid-mediated tigecycline resistance genes, *tet*(X3) and *tet*(X4), in animals and humans in China would pose a foreseeable threat to public health. To illustrate this paradigm shift in tigecycline resistance, here, covering the period 2008-2018, we retrospectively analysed a national strain collection of *Escherichia coli* (n = 2254), obtained from chickens and pigs, in six representative provinces of China. The gene *tet*(X4) was identified in five pig isolates collected in 2016 and 2018 from the provinces of Sichuan (3/15, 2018), Henan (1/25, 2018) and Guangdong (1/28, 2016), but not in the isolates prior to 2016. None of the isolates was detected harbouring *tet*(X3). All *tet*(X4)-positive *E. coli* exhibited high levels of tigecycline resistance (MICs, 16-64 mg/L), and two were confirmed as colistin resistant, harbouring chromosome-borne *mcr-1* gene. The gene *tet*(X4) was detected on a plasmid in all five isolates, whereas a co-location of *tet*(X4) on the chromosome of one isolate was observed. Diverse host strains and novel plasmids related to the *tet*(X4) gene were observed. Our timely findings of the recent emergence of *tet*(X4) gene in food animal support the rapid surveillance and eradication of this gene before it is established.

In late May 2019, He et al. reported the emergence of plasmid-mediated tigecycline resistance due to the presence of the novel *tet*(X3) or *tet*(X4) gene in numerous *Enterobacteriaceae* and *Acinetobacter* isolates in China [1]. An alarming number (as far to 43.3%) of the isolates carried either the *tet*(X3) or *tet*(X4) resistance determinant were identified in animals (pig, chicken and cow) and meat for consumption (chicken and pork) from three provinces surveyed [1]. Of even more concern, *tet*(X4)-positive *Escherichia coli* and *Acinetobacter baumannii* were isolated from secretion samples of inpatients in two Chinese provinces [1]. The two transferable high-level tigecycline resistance genes could severely compromise the efficacy of tigecycline in vivo (mice model), one of the few remaining alternatives for treating complicated infections caused by multidrug-resistant bacteria in human, thus would pose a foreseeable threat to public health as a result of clinical treatment failure [1].

The high detection rates of *tet*(X3) and *tet*(X4) genes in bacteria from animals invoked the researchers [1–3] a speculation that these genes are likely to result from the selective pressure of tetracyclines utilized heavily in animals for decades. To illustrate this paradigm shift in tigecycline resistance in food-producing animals in China, we retrospectively analysed a large strain collection of *E. coli*, obtained from chickens and pigs, in six representative provinces of China from 2008 to 2018.

Based on the China national surveillance programme on antimicrobial resistance (AMR) in zoonotic bacteria, *E. coli* strains collected from chicken cloacal swabs and pig faecal swabs over a span of more than five years in each province were included in the study, resulting a total of 2254 *E. coli* strains isolated in the year between 2008 and 2018 (Figure 1(A)) without any targeted AMR phenotypes. The isolates originated from farms or slaughterhouses located in six provinces from different geographical divisions of China (northeast, east, south and southwest), including Liaoning (*n* = 343, 2010–2018), Shandong (*n* = 250, 2013–2017), Henan (*n* = 299, 2013–2018), Guangdong (*n* = 489, 2008–2018), Sichuan (*n* = 383, 2011–2018) and Shanghai city (*n* = 490, 2010–2018) (Figure 1(B)). The disunity in year distribution of the isolates results from the time difference in joining in the national AMR surveillance programme. All isolates were maintained by China Institute of Veterinary Drug Control in Beijing.

**Figure 1.**
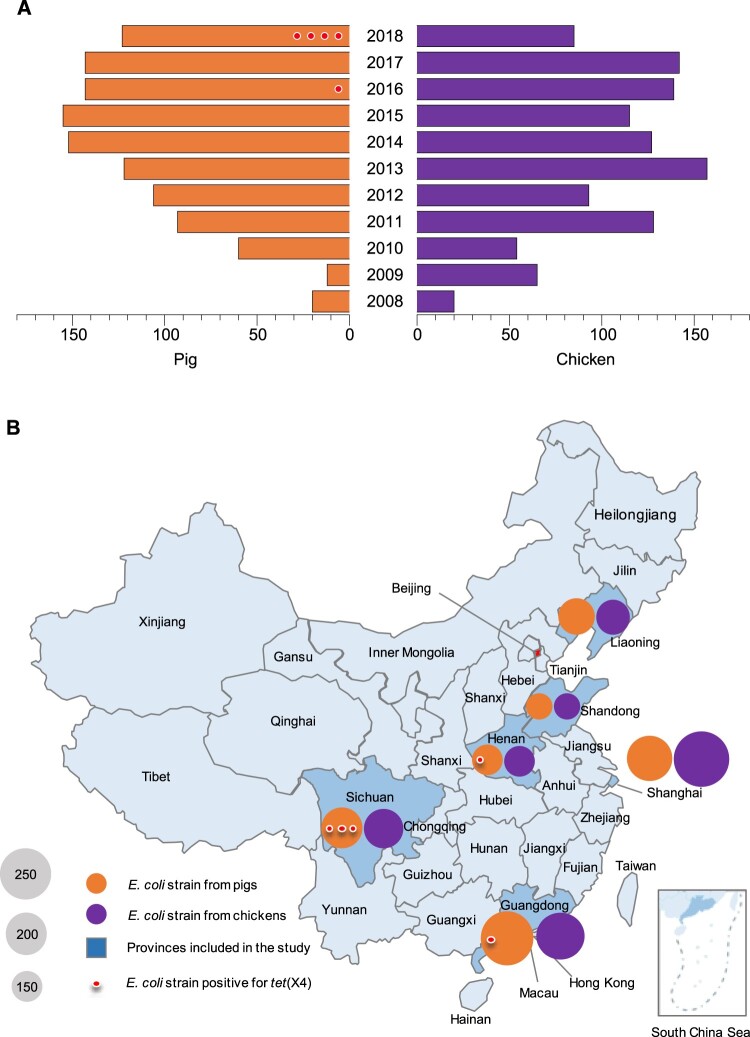
Year (A) and geographical (B) distribution of *E. coli* strains from pigs and chickens included in the study, China, 2008–2018 (*n* = 2254). Red dots indicate the *E. coli* strains positive for *tet*(X4), one dot per isolate.

*E. coli* isolates were subjected to MacConkey plates supplemented with tigecycline (2 mg/mL) and resulting a total of 59 tigecycline-non-susceptible isolates, from which we sought bacteria harbouring the novel tigecycline-resistant genes, *tet*(X3) and *tet*(X4), as described by He et al. [1]. The genome DNA of *tet*(X3)-carrying *A. baumannii* 34AB and *tet*(X4)-carrying *E. coli* 47EC in He’s study were served here as positive controls [1]. In total, *tet*(X4) gene was detected in five pig isolates from the provinces of Sichuan (3/15, 20.0%, 2018), Henan (1/25, 4.0%, 2018) and Guangdong (1/28, 3.6%, 2016). All amplified fragments had 100% nucleic acid sequence homology with the previously described *tet*(X4) gene [1]. None of the isolates was detected harbouring *tet*(X3) in the current strain collection. Neither *tet*(X4) nor *tet*(X3) was detected in isolates prior to 2016. Together with the 2019 reports on the occurrence of *tet*(X4) gene in *E. coli* from food animals, pork, soil and dust samples in China [1–3], our findings suggest the emergence of the *tet*(X4) gene in farm animals in China is a recent event.

Antimicrobial susceptibility test using broth microdilution [4] confirmed all *tet*(X4)-positive *E. coli* exhibited high levels of tigecycline resistance (MICs, 16–64 mg/L) and doxycycline resistance (MICs, 64–128 mg/L), whereas none of these isolates demonstrated resistance to meropenem and cefepime. Moreover, all *tet*(X4)-positive *E. coli* were multidrug-resistant (MDR), in that they were resistant to three or more different classes of antimicrobials and two were confirmed as colistin resistant (SC-P315 and SC-P336) (Table S1). Whole genome sequencing (WGS) of a 150 bp paired-end library of each isolate was conducted using the Illumina HiSeq X Ten System with a minimum of 100-fold coverage. Subsequent stand-alone BLAST analysis against ResFinder (29 April 2019) [5] identified a set of resistance determinants from each draft assembly, including but not limited to genes conferring resistance to tetracyclines [*tet*(A), *tet*(B), *tet*(M) and *tet*(X4)], beta-lactams (*bla*_CMY-2_ and *bla*_TEM-1B_), quinolones (*oqxA* and *oqxB*), aminoglycosides (*aac(3)-IId*, *aac(3)-Via* and *aadA1*), phenicol (*floR*, *cmlA1*) and colistin (*mcr-1*) (Table S1). Of concern, the two colistin-resistant *tet*(X4)-positive *E. coli* strains co-harbouring *mcr-1*, rendering co-resistance to tigecycline and colistin, the remaining last-resort antibiotics for treating serious infections. A much comprehensive study detected samples of pigs, chickens, soil and dust illustrated a wide distribution of *tet*(X4)-positive *E. coli* co-harbouring *mcr-1* (22.5%, 9/40) in China [2]. In this context, it is worrisome to speculate that the dissemination of these extensively drug-resistant bacteria represents a serious threat to human and animal health.

*In silico* multilocus sequence typing (MLST) clustered the five *tet*(X4)-positive *E. coli* strains into four distinct STs, which were three known STs, ST2345 (HN-PA19), ST4535 (GD-P230), ST4541 (SC-P337), along with one new type assigned as ST9772 (SC-P315 and SC-P336). Sequence mapping [6] of the two ST9772 genomes to a fully sequenced *E. coli* reference (NC_010498) identified 52 substitution single nucleotide polymorphisms (SNPs) between the 2 isolates, indicating 2 different lineages.

S1-pulsed-field gel electrophoresis (PFGE) profiling showed that three of the isolates possessed multiple plasmids differing in size, whereas the genomes of the remaining two ST9772 isolates were over-digested by S1 nuclease after multiple attempts (Figure S1). Subsequent Southern hybridization analysis located the *tet*(X4) gene on the plasmid of the three isolates, in sizes of approximately 230-kb (GD-P230), 180-kb (HN-PA19) and 170-kb (SC-P337), respectively. Of interest, a co-location of *tet*(X4) gene on the chromosome of SC-P337 was observed (Figure S1). To further characterize the *tet*(X4) gene in the two *mcr-1*-positive isolates, long-read Nanopore MinION sequencing, followed by hybrid de novo assembly combining short-read Illumina reads was performed [7]. Sequence analysis identified the gene *tet*(X4) on the plasmids in sizes of 8581 bp (pSC-P315) and 14,433 bp (pSC-P336), while the *mcr-1* gene locate on the chromosome in both of the two isolates. Annotations and blast analysis indicate that pSC-P315 shared similar plasmid backbone (60% query coverage and 99% average sequence identity) against pSC-P336. Incompatibility typing of the two *mcr-1*-carrying plasmids using PlasmidFinder [8] showed no hit found, indicates they are novel plasmids currently non-typable. Taken together with the diverse host strains and promiscuous plasmid types related to the *tet*(X4) gene ever observed [1–3], the transmission of this gene among different sectors (animal, human and the environment) will be further complicated.

We subsequently analysed the detailed genetic context of the *tet*(X4)-harbouring contigs (Figure S2). As observed from the original *tet*(X4)-harbouring plasmid p47EC [1], the ISV*sa3* element franking the *tet*(X4) gene were conserved in all five contigs, while the downstream region of *tet*(X4)-IS*Vsa3* observed in p47EC, composing genes coding an ABC transporter, two hypothetical protein and a truncated ISV*sa3* was missing in the corresponding region of SC-P336, SC-P337 and SC-P315. Phenicol resistance gene *floR* was found downstream of *tet*(X4)-IS*Vsa3* in three of the five isolates, which suggested that the tigecycline resistance may be co-selected by other antimicrobial resistances.

In this study, covering the period 2008–2018, we retrospectively demonstrated the emergence of the novel plasmid-mediated tigecycline resistance gene *tet*(X4) in farm animals in China is a recent event. This is likely a different scenario reminiscent of the well-documented colistin resistance gene *mcr-1*, which emerged early in animals in the 1980s (outbreak in around 2009) [9] and apparently too late to eradication on its first report ∼40 years later in 2015 [10]. Tigecycline has never been licenced for animal husbandry, while for decades, tetracycline agents of the earlier generations have been widely used in veterinary medicine in China [11] and also many other countries, particularly the Europe settings [12] and USA [13]. This presumably provides a continuously selective pressure for the recent paradigm shift in tigecycline resistance, and may also for the subsequent worldwide disseminations, as exemplified by *mcr-1* [14]. Given the fact of extensively international trade of food-producing animals and products derived from them [15], our timely findings strongly recommend the rapid surveillance and eradication of these genes before it is established, particularly in countries or regions with heavily tetracyclines consumptions.

## Supplementary Material

Supplemental Material
