# Supplementary material for: Plasmid-mediated tigecycline-resistant gene tet(X4) in Escherichia coli from food-producing animals, China, 2008–2018
Source: Emerg Microbes Infect. 2019 Oct 21;8(1):1524–7. doi: 10.1080/22221751.2019.1678367 (PMC6818123; doi:10.1080/22221751.2019.1678367)
Supplement: Supplemental Material [file TEMI_A_1678367_SM5472.doc]

**Table S1.** Phenotypic and genotypic characteristics of *tet*(X4) positive *E. coli* isolated in the province of Guangdong, Henan and Sichuan, China, 2016 and 2018.

| Isolates | Year of isolation | Origin | STs | Tigecycline MIC (mg/L) | Antimicrobial resistance profiles ^b^ | Resistance determinants identified based on WGS ^c^ |
| --- | --- | --- | --- | --- | --- | --- |
| GD-P230 | 2016 | Pig | ST4535 | 32 | AMP-FFC-SXT-DOX-TGC | *aadA24,* *strA*, *strB, sul1*, *sul2*, *QnrS1*, *bla*_TEM-1B_, *floR*, *dfrA1*, *tet*(A), *tet*(X4) |
| HN-PA19 | 2018 | Pig | ST2345 | 16 | AMP-FFC-GEN-SXT-DOX-TGC | *aadA2*, *aac(3)-IId*, *aph(3')-Ia*, *sul2*, *sul3*, *QnrS1*, *bla*_TEM-1B_, *cmlA1*, *floR*, *dfrA12*, *lnu*(F), *tet*(A), *tet*(M), *tet*(X4) |
| SC-P315 | 2018 | Pig | ST9772 ^a^ | 16 | AMP-AMC-AZM-CIP-FFC-GEN-SXT-ZOX-COL-DOX-TGC | *aac(3)-VIa*, *aadA1*, *sul1*, *erm(42)*, *oqxA*, *oqxB*, *bla*_CMY-2_, *mcr-1*, *tet*(B)*, tet*(X4) |
| SC-P336 | 2018 | Pig | ST9772 ^a^ | 16 | AMP-AMC-AZM-CIP-FFC-GEN-SXT-ZOX-COL-DOX-TGC | *aac(3)-VIa*, *aadA1*, *sul1*, *erm(42)*, *oqxA*, *oqxB*, *bla*_CMY-2_, *floR*, *mcr-1*, *tet*(B)*, tet*(X4) |
| SC-P337 | 2018 | Pig | ST4541 | 64 | AMP-AMC-AZM-FFC-GEN-DOX-TGC | *aac(3)-VIa*, *aac(3)-IId*, *aadA1*, *sul1*, *erm(42)*, *oqxA*, *oqxB*, *bla*_CMY-2_, *bla*_TEM-1B_, *tet*(A)*, tet*(X4) |

a: New STs assigned for the first time in this study.

b: Tigecycline resistance breakpoint tentatively set at MIC≥8 mg/L.

c: The gene *tet*(X4) was identified based on stand-alone blast, the other acquired antimicrobial resistance genes were identified based on ResFinder database (2019-04-26).

AMC: amoxicillin-clavulanate; AMP: ampicillin; AZM: aztreonam; CIP: ciprofloxacin; COL: colistin; DOX: doxycycline; FFC: florfenicol; GEN: gentamicin; SXT: trimethoprim/sulfamethoxazole; TGC: tigecycline; AXO: ceftriaxone; WGS, whole genome sequencing.

**Figure S1. S1-PFGE and Southern hybridisation analysis of *tet*(X4)-positive plasmids harboured by E. coli isolated from pigs in the province of Guangdong, Henan and Sichuan, China, 2016 and 2018.**

SH: Southern hybridization; M: marker; N: *E. coli* isolates negative for *tet*(X4); The genome of GD-P230, N, HN-PA19 and SC-P337 in plugs were well digested with 10 U S1 nuclease at 37 °C for 15 min, while the genome of SC-P315 and SC-P336 were over-digested with 5 U (a), 1.25 U (b) and 0.6 U (c) S1 nuclease at 37 °C for 15 min.

**Figure S2.** **Schematic representation and comparison of the genetic context of *tet*(X4)- harbouring contigs in the five isolates in the province of Guangdong, Henan and Sichuan, China, 2016 and 2018.**

Arrows indicate the directions of genes, colours differing genes of various functions (red: antibiotic resistance associated gene; yellow: transfer associated gene; green: integrase, recombinase or transposase; grey: gene of other function). Regions of >99.0% nucleotide sequence identity are indicated by shading.
